# Gods are watching and so what? Moralistic supernatural punishment across 15 cultures

**DOI:** 10.1017/ehs.2023.15

**Published:** 2023-05-12

**Authors:** Theiss Bendixen, Aaron D. Lightner, Coren Apicella, Quentin Atkinson, Alexander Bolyanatz, Emma Cohen, Carla Handley, Joseph Henrich, Eva Kundtová Klocová, Carolyn Lesorogol, Sarah Mathew, Rita A. McNamara, Cristina Moya, Ara Norenzayan, Caitlyn Placek, Montserrat Soler, Tom Vardy, Jonathan Weigel, Aiyana K. Willard, Dimitris Xygalatas, Martin Lang, Benjamin Grant Purzycki

**Affiliations:** 1 Aarhus University, Aarhus, Denmark; 2 Department of Psychology, University of Pennsylvania, Philadelphia, Pennsylvania; 3 Department of Psychology, University of Auckland, Auckland, New Zealand; 4 Max Planck Institute for the Science of Human History, Jena, Germany; 5 College of DuPage, Glen Ellyn, Illinois, USA; 6 Wadham College, University of Oxford, Oxford, UK; 7 Arizona State University, Phoenix, Arizona, USA; 8 Department of Human Evolutionary Biology, Harvard University, Cambridge, Massachusetts, USA; 9 LEVYNA, Masaryk University, Brno, Czech Republic; 10 Washington University in St Louis, St. Louis, Missouri, USA; 11 School of Psychology, Victoria University of Wellington, Wellington, New Zealand; 12 Department of Anthropology, University of California Davis, Davis, California, USA; 13 Department of Psychology, University of British Columbia, Vancouver, British Columbia, Canada; 14 Department of Anthropology, Ball State University, Muncie, Indiana, USA; 15 Ob/Gyn and Women's Health Institute Cleveland Clinic, Cleveland, Ohio, USA; 16 Department of International Development, London School of Economics, London, UK; 17 Haas School of Business, University of California Berkeley, Berkeley, California, USA; 18 Brunel University, London, UK; 19 Department of Anthropology, University of Connecticut, Storrs, Connecticut, USA; 20 Department of Psychological Sciences, University of Connecticut, Storrs, Connecticut, USA

**Keywords:** Behavioural economics, cognitive anthropology, cultural evolutionary psychology, evolutionary and cognitive science of religion, free-list

## Abstract

Psychological and cultural evolutionary accounts of human sociality propose that beliefs in punitive and monitoring gods that care about moral norms facilitate cooperation. While there is some evidence to suggest that belief in supernatural punishment and monitoring generally induce cooperative behaviour, the effect of a deity's *explicitly postulated* moral concerns on cooperation remains unclear. Here, we report a pre-registered set of analyses to assess whether perceiving a locally relevant deity as moralistic predicts cooperative play in two permutations of two economic games using data from up to 15 diverse field sites. Across games, results suggest that gods’ moral concerns do not play a direct, cross-culturally reliable role in motivating cooperative behaviour. The study contributes substantially to the current literature by testing a central hypothesis in the evolutionary and cognitive science of religion with a large and culturally diverse dataset using behavioural and ethnographically rich methods.

## Graphical abstract

**Figure:**
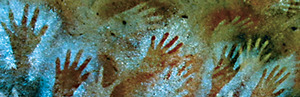

**Social media summary:** A watchful and punitive god can make people more cooperative, but how does a deity's perceived moral concerns play into this phenomenon?

## 1. Introduction

Scholars of religion have long contemplated how religious appeals, beliefs and rituals are implicated in human cooperation (Durkheim, 1912; Ellwood, 1918; Evans-Pritchard, 1965; Lang, 1909; Malinowski, 1936; Rappaport, 1968; Wallace, 1966; for recent reviews, see Bendixen et al., 2023b; McKay and Whitehouse, 2014; Purzycki and McKay, 2023). Recent psychological and cultural evolutionary accounts of human sociality have proposed that beliefs about ‘moralising’ deities – that is, punitive and monitoring gods and spirits believed to be concerned with violations of inter-personal norms (Purzycki and McNamara, 2016) – foster cooperative relationships (Johnson, 2016; Shariff and Norenzayan, 2007) and help societies increase in size and complexity (Norenzayan et al., 2016; Schloss and Murray, 2011).

While there is some evidence to support a relationship between supernatural punishment beliefs and cooperation, individual-level studies remain inconclusive with regards to establishing a link between the *moral content* of supernatural punishment beliefs specifically and cooperative behaviour (Bendixen et al., 2023b). In this study, across 15 diverse field sites and two economic games, we assess the importance of moral content in moralistic supernatural punishment beliefs and its role in motivating cooperative behaviour.

### 1.1. Supernatural moral punishment and cooperation: A tour of the evidence

A family of theories holds that belief in moralising deities facilitates cooperation through fear that moral norm violations are supernaturally monitored and sanctioned. In turn, by harnessing psychological systems responsible for cooperative behaviour, such beliefs are ostensibly one mechanism that can help resolve problems associated with collective action and cooperation (Schloss and Murray, 2011; Purzycki and McKay, 2023). As such, morally concerned gods in particular are posited to help scale up societies in response to social conflict (Caluori et al., 2020; Skali, 2017) and ecological threats (Hayden, 1987). Society-level analyses based on data coded from ethnographic material indeed suggest that moralistic supernatural punishment beliefs are positively associated with a society's size and political stratification (e.g. Roes and Raymond, 2003; Swanson, 1960; Watts et al., 2015) and with environmental stressors (e.g. Botero et al., 2014; Jackson et al., 2021; Skoggard et al., 2020; Snarey, 1996).

However, database studies of coded ethnographic sources suffer from a variety of biases, including systematic missingness in focal variables (Beheim et al., 2021; Purzycki et al., 2022a), non-representative sampling of cultural groups and antiquated data coding schemes (e.g. whether a moralising god is also by necessity a *high god*, that is a creator deity; see Lightner et al., 2022; Purzycki and McKay, 2023; Watts et al., 2015). Further, surveys of the ethnographic record (e.g. Boehm, 2008; Bendixen and Purzycki, 2023a; Purzycki and McNamara, 2016; Rossano, 2007; Swanson, 1960) as well as recent individual-level ethnographic inquiries (e.g. Bendixen et al., 2023a; Purzycki, 2011, 2013, 2016; Purzycki et al., 2022c; Singh et al., 2021; Townsend et al., 2020) strongly indicate that notions of supernatural punishment of moral norm violations are common even in smaller-scale societies, calling into question the reliability of databases that suggest otherwise (Lightner et al., 2022; Purzycki and McKay, 2023).

Studies using individual-level data have found mixed evidence of a causal relationship between moralistic supernatural punishment beliefs and increased cooperation. For instance, Ge et al. (2019) failed to find a clear association between belief in supernatural punishment and charitable donations in cross-community economic games with a culturally diverse sample. Conversely, Townsend et al. (2020) found that, among the Ik of Uganda, reminding participants about the possibility of supernatural punishment induced higher allocation of money to a needy and anonymous co-community member than in a control condition. Survey (e.g. Atkinson and Bourrat, 2011; White et al., 2019) and experimental (e.g. Shariff et al., 2016; Yilmaz and Bahçekapili, 2016) studies conducted primarily in industrialised societies likewise tend to find positive relationships between beliefs in supernatural punishment and/or reward and various indices of cooperation (cf. Bloom, 2012; Galen, 2012). Across 15 field sites and two waves of data collection, Lang et al. (2019) found a small but robust association between ratings of deities as punitive and monitoring and non-selfish coin allocations in two anonymous economic games, supporting the notion that moralising religions can indeed contribute to an expansion of cooperative circles. Critically, however, in Lang et al. (2019) ratings of deities as morally concerned – specifically, a three-item ‘moral interest scale’ on the extent to which a deity cares for punishing theft, lying and murder (see also Purzycki et al., 2016b) – did *not* consistently predict cooperative behaviour, casting doubt on the extent to which item scales of this sort reliably reflect culturally relevant models of gods’ concerns (Purzycki et al., 2022c).

Taken together, while supernatural punishment and monitoring generally seem to induce cooperative behaviour, the effect of a deity's *explicitly postulated* moral concerns on cooperation remains unclear. It is central to theory, however, since many deities across cultures are attributed with punitive concerns that seem to directly correspond to local coordination problems, such as territoriality, ecological management, resource distribution and, crucially, breaches of moral norms (Bendixen and Purzycki, 2023a; Bendixen et al., 2023a; Purzycki and McNamara, 2016; Purzycki et al., 2022c). As such, both *measuring* these punitive concerns with culturally appropriate methods and *determining* whether they guide individuals’ cooperative behaviour would help clarify the role that supernatural appeals and beliefs might have played in human societies, past and present (Bendixen et al., 2023b). Using both behavioural and ethnographically rich methods, the present study investigates whether or not gods’ explicitly postulated moral concerns predict cooperative behaviour across up to 15 diverse field sites.

## 2. Hypotheses, data and methods

### 2.1. The present study

Here, we report a set of pre-registered analyses to assess the importance of moral content in moralistic supernatural punishment beliefs and its role in motivating cooperative behaviour. Specifically, we assess whether *free-listing* a locally relevant moralistic deity as angered by immorality predicts cooperative play in two permutations of each of two economic games. In the supplementary materials (Section S4) we outline all deviations from the pre-registration protocol; deviations were few and minor.

As an ethnographic technique, free-listing reflects cognitive and cultural models of the target topic, to the extent that this topic is relevant in the local context (Quinlan, 2017). Free-listing might therefore be a more appropriate measure of a deity's degree of moral concern than pre-fabricated item scales used in previous studies. In fact, a recent cross-cultural methodological analysis (Bendixen and Purzycki, 2023b) failed to find clear evidence of within-subject agreement between the three-item ‘moral interest scale’ from Lang et al. (2019) and Purzycki et al. (2016b) and a corollary free-list task, hinting at a dissociation between these two instruments. While free-lists are more often used for descriptive or exploratory purposes (e.g. informing item scale construction), here we leverage free-list data as a predictor variable in a series of multilevel regression models. This arguably allows for higher resolution and a more ethnographically rich and relevant assessment of individuals’ cultural beliefs and their behavioural implications, compared with forced item responses.

In addition, the studies reviewed above suggest that we should distinguish between a deity's tendency to punish and monitor inter-human behaviour more broadly from a deity's *explicitly postulated* moral concerns. Here, we investigated whether attributing both moral concern *and* broad capabilities for punishment and monitoring to a deity (i.e. interaction effects) facilitates cooperative in economic game play to a greater extent compared with these attributes in isolation (i.e. additive effects). That is, does the perceived scope of a deity's capabilities for monitoring and punitiveness moderate the impact on cooperative behaviour of the moral concerns attributed to that deity? Our key hypotheses are as follows:

**H_1_:** The more an individual claims their god cares about morality, the more they will exhibit cooperative behaviour.

**H_2_:** The more an individual claims their god knows, punishes *and* cares about morality, the more they will exhibit cooperative behaviour.

In our analyses, cooperation is measured as coin allocations in two different economic games and two permutations of each game (Section 2.2). We operationalise *morality* according to a pre-specified category of relevant human behaviours and attributes as they appear in our coded free-list data on a locally relevant god's concerns (Section 2.3). We operationalise supernatural *punishment* and *knowledge breadth* according to two item scales and hold relevant covariates constant (Section S3.1). In the context of our study, then, H_1_ entails that free-listing a locally relevant deity as moralistic should predict increased probability of impartial coin allocations. H_2_ entails that free-listing a locally relevant deity as moralistic *while also* rating that same deity as punitive *and* omniscient (i.e. a three-way interaction) predicts increased probability of impartial coin allocations.

We first lay out our methods and data sources (Section 2; see also Section S3), a summary of the main statistical analyses (Section 2.4) and then report results (Section 3). Finally, we discuss key implications of our analysis (Section 4). In the supplementary materials, Sections S1–S3 provide more detail on our causal assumptions, statistical models and data. Section S4 documents deviations from the pre-registered statistical protocol.

The data come from the Evolution of Religion and Morality Project (Lang et al., 2019; Purzycki et al., 2016a). The full dataset consists of two waves of data collection across a total of 15 field sites (Table 1). As such, while the present study is novel, parts of the dataset have already been analysed – for site-specific and omnibus reports, see: Tanna, Vanuatu (Atkinson, 2018; Vardy and Atkinson, 2022), Lovu, Fiji (Willard, 2018), Mauritius (Klocová et al., 2022; Xygalatas et al., 2018), Pesqueiro, Brazil (Cohen et al., 2018), Tyva Republic (Purzycki and Kulundary, 2018), Yasawa, Fiji (McNamara and Henrich, 2018; McNamara et al., 2021), Hadza, Tanzania (Apicella, 2018; Stagnaro et al., 2022), Sursurunga, Papua New Guinea (Bolyanatz, 2022), Mysore, India (Placek and Lightner, 2022), Cachoeira, Brazil (Soler et al., 2022), Kananga, D. R. Congo (Kapepula et al., 2022) and omnibus (Baimel et al., 2022; Bendixen et al., 2023a; Lang et al., 2019; Purzycki and Lang, 2019; Purzycki et al., 2016b, 2018, 2022c; Vardy et al., 2022).

**Table 1.** Selected moralistic deities, primary economy, and cultural group of the anonymous DISTANT recipient in the two economic games for each field site. Game-specific sample sizes: RAG SELF, *N* = 1033; RAG LOCAL, *N* = 1028; DG SELF, *N* = 1077; DG LOCAL, *N* = 1066.

Site	Economy	Moralistic deity	DISTANT recipient
Cachoeira, Brazil	Wage labour	Christian God	Candomblé
Coastal Tanna	Horticulture/hunting	Christian God	Christian
Hadza	Hunting	*Haine*	Hadza
Huatasani, Peru	Agro-pastoralism	Christian God	Catholic
Inland Tanna	Horticulture/hunting	*Kalpapen*	Kastom
Kananga, DRC	Wage labour	Christian God	Non-Luluwa Christian
Lovu, Fiji	Wage labour	Shiva	Hindu
Marajó, Brazil	Wage labour	Christian God	Christian
Mauritius	Wage labour	Shiva	Hindu
Mysore, India	Wage labour/farming	Shiva	Hindu
Samburu, Kenya	Herding/wage labour	Christian/traditional (*Nkai*)	Christian Samburu
Sursurunga, Papua New Guinea	Horticulture	Christian God (*Ka'l’au*)	Christian Sursurunga
Turkana, Kenya	Pastoralism	Christian God (*Akuj*)	Christian Turkana
Tyva Republic	Wage labour/herding	*Buddha–Burgan*	Buddhist
Yasawa, Fiji	Fishing/farming	Christian God	Hindu

RAG, Random Allocation Game; DG, Dictator Game.

### 2.2. Economic games

Our focal outcome measure of cooperation is coin allocations in two economic games, the Random Allocation Game (RAG) and the Dictator Game (DG). The participants played two permutations of each game: SELF vs. DISTANT (i.e. coins are either allocated to the participant themselves or an anonymous, geographically distant co-religionist) and LOCAL vs. DISTANT (i.e. coins are either allocated to a local co-religionist or a distant co-religionist).

Two further conditions were employed in the full protocol: SELF vs. OUTGROUP (i.e. coins are either allocated to the participant themselves or an outgroup member), and DISTANT vs. OUTGROUP (i.e. coins are either allocated to a distant co-religionist or an outgroup member). For the present study, we focus exclusively on the SELF vs. DISTANT and LOCAL vs. DISTANT conditions, since the OUTGROUP conditions entail distinct theoretical predictions (cf. Lang et al., 2019) that are outside the scope of our current aims.

The RAG is a simple economic game experiment designed to measure impartial rule-following (Hruschka et al., 2014; Jiang, 2013). In our RAG, participants were endowed with 30 coins, a fair die with two outcomes (e.g. black and white) and two cups each designated as the cup of two different recipients, a cup for SELF or LOCAL and another cup for DISTANT depending on condition (see just above). For each coin, participants were asked to think of the recipient to whom they wish to allocate the coin and then roll the die. If the die lands with a particular outcome (e.g. black), then the participant can allocate the coin to the wished-for recipient; otherwise, the coin goes in the opposite cup. Participants know that we distribute the money according to the descriptions on the cups. However, since the identities of the actual recipients (other than SELF) are never revealed and participants play alone, they are in a position to place the coins in whichever cup they want. Since we expect the coin allocations to be binomially distributed with a fair die, systematic deviance from this assumption is interpreted as increased (or decreased) rule-breaking in favour of players or their in-group.

**Figure 1.**
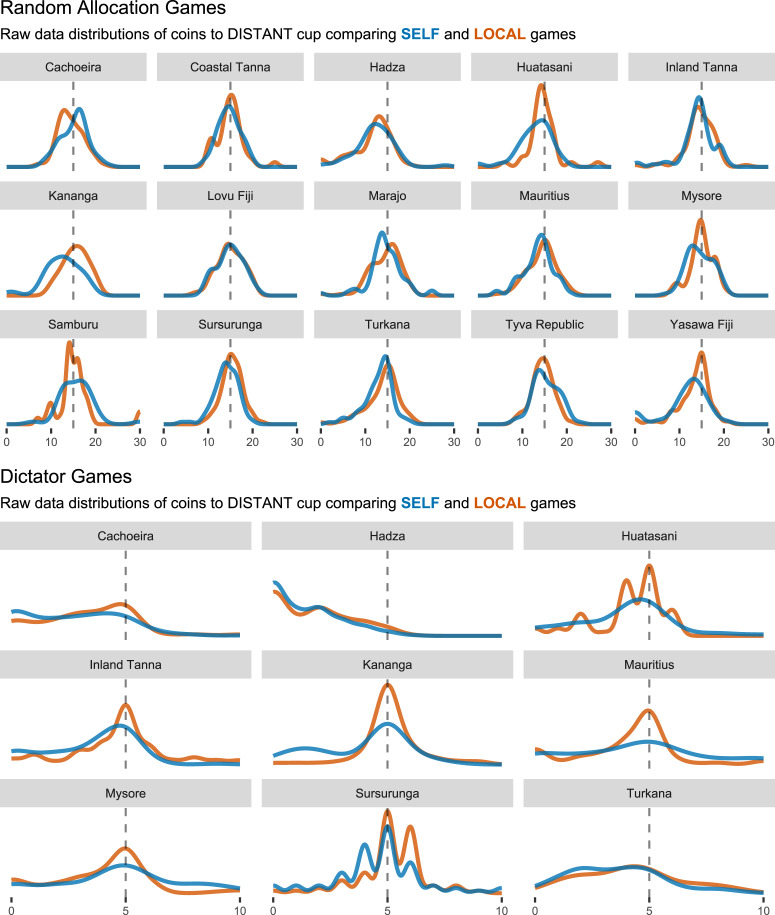
Raw data distributions of Random Allocation Games (RAGs, top) and Dictator Games (DGs, bottom). Densities are number of coins allocated to the DISTANT cup, the focal outcome variables in our analyses, across games and sites. The dashed lines represent the mid-points of the endowment for each game (15 coins for the RAGs, five coins for the DGs).

In the DG, participants are endowed with a stack of coins (10 in this case) and are simply asked to allocate it between two individuals (SELF vs. DISTANT or LOCAL vs. DISTANT, depending on game condition) however they like. As with the RAG, the DG is a measure of self/in-group partiality. Playing two different games with the same sample ensures that any result is not isolated to a particular game set-up. Figure 1 shows the raw data distributions of coins allocated to the DISTANT cup across sites and games. Informally and overall, we would expect that participants allocate less coins to the DISTANT cup in the SELF games (i.e. the blue densities), a pattern that we indeed find in most – but not all – sites.

Across games and conditions, the combined total stakes were set at a local daily wage with a show-up fee of ~25% of the local daily wage. The economic games were played at the outset of the full protocol and the order in which the games were played was counterbalanced.

### 2.3. Free-listing

Free-listing involves simply asking people to list their associations on some topic, in this case what angers a locally relevant moralistic deity. The earlier an item is listed, the more likely it is to appear in the lists of participants drawn from the same cultural milieu (e.g. Bendixen et al., 2023a; Purzycki, 2016). Hence, free-listing measures both *cognitive* and *cultural* models.

As part of the main study protocol, participants were asked to freely list what they thought angered a locally important ‘moralistic deity’ (i.e. a deity that was pre-selected locally based on ethnographic background interviews to be concerned with inter-personal behaviour; see Table 1). The free-list data were subsequently thematically coded by two pairs of independent coders, one pair for each wave, according to a top-down, twelve-category coding rubric (our ‘general codes’) drawn from Purzycki and McNamara (2016). According to the general coding rubric, ‘Morality’ was coded as *generalised b*e*haviours that have a benefit or cost to other people* (*e.g. hurting, being generous, sharing, etc.)* (Bendixen et al., 2023a).

**Figure 2.**
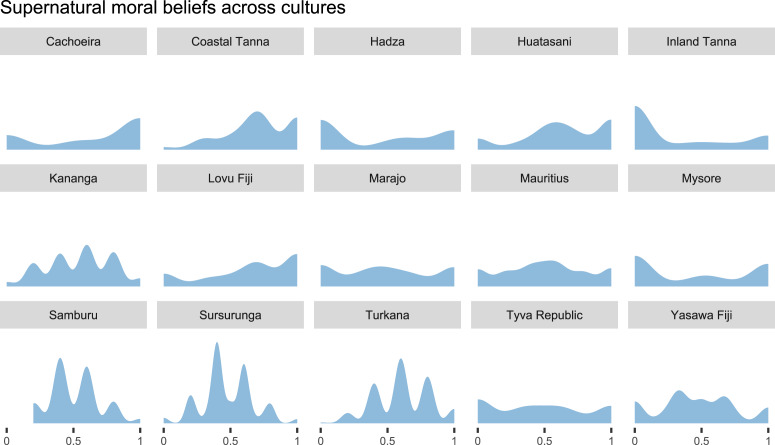
Cross-cultural distributions of supernatural moral beliefs. Densities are proportions of moral items in free-lists on what angers focal deities, the focal predictor variable in our main analyses. Note that the overlap between the plotted data and the samples used for analyses is imperfect, since some participants responded to the free-list task but did not complete one or more of the economic games.

In our analysis, we use free-list responses to predict game behaviour in a series of Bayesian multilevel regression models. As we are interested in measuring the extent to which individuals ascribe moral concern to their deities, we use the *proportion of moral items* in each participant's lists as our focal predictor (see Section S3.3). Figure 2 plots the cross-cultural distributions of the proportion of free-listed moral items across sites in our sample.

Free-list data were cleaned (see Section S3.2) and then processed with the AnthroTools package (Jamieson-Lane and Purzycki, 2016; Purzycki and Jamieson-Lane, 2016) for R (R Core Team, 2021). Note that in the full study protocol, in addition to free-listing what they thought angered the locally important ‘moralistic deity,’ participants were also asked to list the kinds of things that please the target deity. Participants were also prompted to list what angers and pleases a ‘local deity’ (i.e. a deity that is locally relevant but pre-selected to be less moralistic, punitive, and knowledgeable) and the police (for an empirical report of the wave 1 free-list data, see Bendixen et al., 2023a). Both kinds of deities were selected for each site on the basis of preliminary ethnographic interviews. For the present study, we focus on the ‘moralistic deities’, since for each field site the frame of the experimental game was explicitly about the religious traditions of those deities. We focus on what *angers* these deities, since we're interested in their punitive aspects.

### 2.4. Statistical analyses

We analysed the RAG with a binomial model (see Section S2.1), the DG with an ordered categorical model (Section S2.2) and modelled the SELF and LOCAL games in separate model sets. All models are Bayesian multilevel regression models with weakly regularising prior settings. In the pre-registration, we ran the models on synthetic data to ensure that the models in fact recover key parameters in an ideal scenario under the assumed data-generating process. While we assume no systematic missingness conditional on our covariates, our statistical models employ full Bayesian imputation of missing covariates (McElreath, 2020) in order not to discard data unnecessarily. Key model diagnostics and posterior predictive checks were generally acceptable and are reported in the online supplementary materials (Section S5).

For H_1_ the key parameter is that of free-listed morality on coin allocation. To assess H_2_ for each of the outcomes we fit and compare two different models: (1) the theoretically informed ‘interaction model’ including a three-way interaction term between punitiveness, knowledge breadth and free-listed morality as well as its two-way interaction components and main effects; and (2) an ‘additive model’, which excludes all interaction terms but retains the main effects. The additive model then serves as a ‘null model’ to be contrasted with the theoretically informed interaction model. See Sections S1 and S2 for further detail on our causal assumptions and statistical models.

To assess H_1_, our estimand across analyses is *the marginal contrast in posterior predicted probabilities of allocating coins to the DISTANT cup between free-listing* ‘*Morality*’ *vs. not free-listing* ‘*Morality*’, obtained using *g*-computation (for further methodological details, see Section S2.3). We summarise the contrasts by their posterior mean and 95% highest posterior density interval (HPDI), the narrowest region of the posterior distribution containing 95% of the parameter estimates. In the RAGs, if the contrast is positive, there's an on-average higher probability of allocating a coin to the DISTANT cup, when listing only moral content (*M* = 1) among the focal deity's concerns, compared with not free-listing any moral content (*M* = 0). Likewise in the DGs, for a given number of coins between 0–10, if the contrast is positive, there's an on-average higher probability of allocating that number of coins to the DISTANT cup, when listing only moral content (*M* = 1) among the focal deity's concerns, compared with not free-listing any moral content (*M* = 0). Our estimates have a causal interpretation to the extent that relevant identification assumptions are satisfied (see Section S1).

Model code and data were prepared with the rethinking package (McElreath, 2020) for R and fit with Stan via cmdstanr (Carpenter et al., 2017; Gabry et al., 2022). See the online supplementary materials (Section S5) for a complete list of R packages, their dependencies and version numbers used for this study.

## 3. Results

### 3.1. Do gods’ moral concerns predict cooperation?

**Figure 3.**
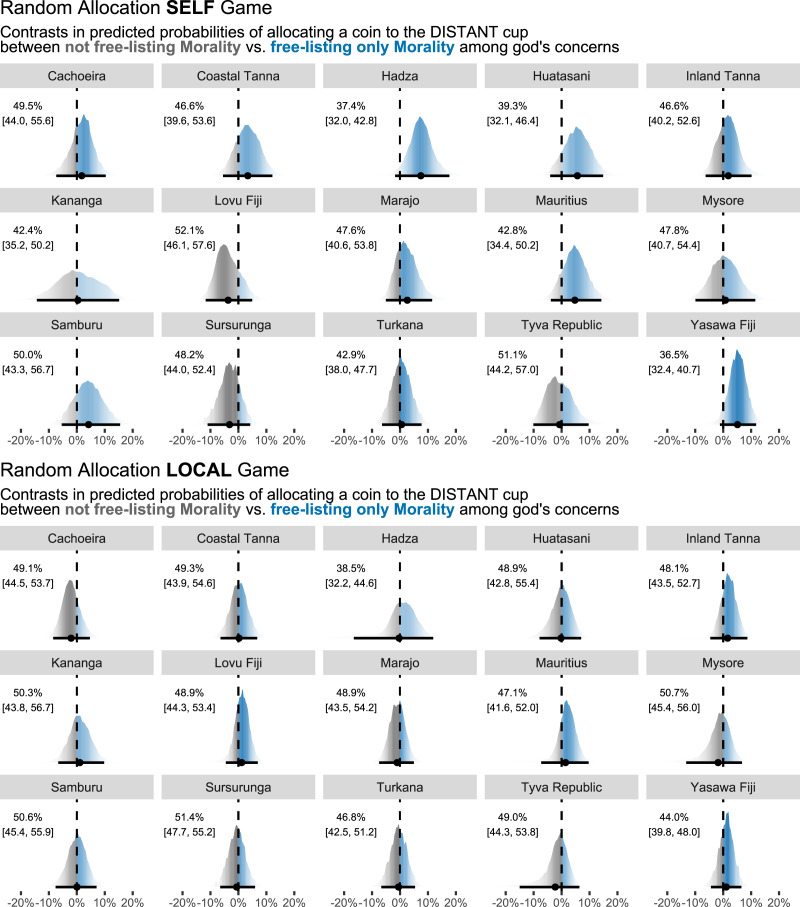
Random Allocation Games, SELF (top) and LOCAL (bottom). Marginal contrasts in posterior predicted probabilities of allocating a coin to the DISTANT cup (in percentage points). If the contrast is positive (blue), there's an on-average higher probability of allocating a coin to the DISTANT cup, when free-listing only moral content (*M* = 1) among the focal deity's concerns. If the contrast is negative (grey), there is an on-average higher probability of allocating a coin to the DISTANT cup, when not free-listing moral content (*M* = 0) among the focal deity's concerns. Printed numbers are site-specific posterior predicted probabilities of a coin allocation to DISTANT, when *M* = 0 with 95% highest posterior density intervals (HPDIs) in brackets. 0% (dashed line) means no difference. Posterior means and 95% HPDIs in black. Colour gradients reflect posterior mass. Distributions are normalised.

For H_1_, while we find support for the predicted effects in some sites, overall moral concerns do not play a cross-culturally reliable role in motivating cooperative behaviour. Figure 3 illustrates the contrast in posterior predicted probabilities of allocating a coin to the DISTANT cup in the RAGs (upper panel, SELF game; lower panel, LOCAL game) between perceiving the focal deity as maximally moralistic (i.e. free-listing only moral responses, *M* = 1) vs. not moralistic at all (i.e. not free-listing any moral responses, *M* = 0). The printed numbers are site-specific mean probabilities of a coin allocation to DISTANT, when *M* = 0 (with 95% HPDIs in brackets), whereas the distributions (summarised by their mean and 95% HPDIs in black points and lines) are the contrasts when *M* = 1. Recall that when the contrast is positive (i.e. the blue parts of the distributions), it implies that, on average, participants were more likely to allocate a coin to the DISTANT cup, if they free-listed the focal deity as moralistic compared with not free-listing moral responses. When the contrast is negative (i.e. the grey parts of the distributions), the opposite relationship is implied on average.

For the SELF RAG, several sites trend in the predicted positive direction (particularly Hadza, Huatasani, Mauritius, and Yasawa) such that the bulk of the posterior masses are positive. At these sites, people are on average around 5 percentage points more likely to allocate a coin to the DISTANT cup if they free-list their focal deity as maximally moralistic, although the estimates are also consistent with little-to-no effect, as indicated by the HPDIs. Other sites have posterior means close to zero or slightly below. Overall, these results suggest the absence of a reliable, cross-cultural effect. The same inference can be drawn for the LOCAL RAG, although here a null effect is arguably more evident, in that the posterior mass is in almost all cases centred on or close to zero.

**Figure 4.**
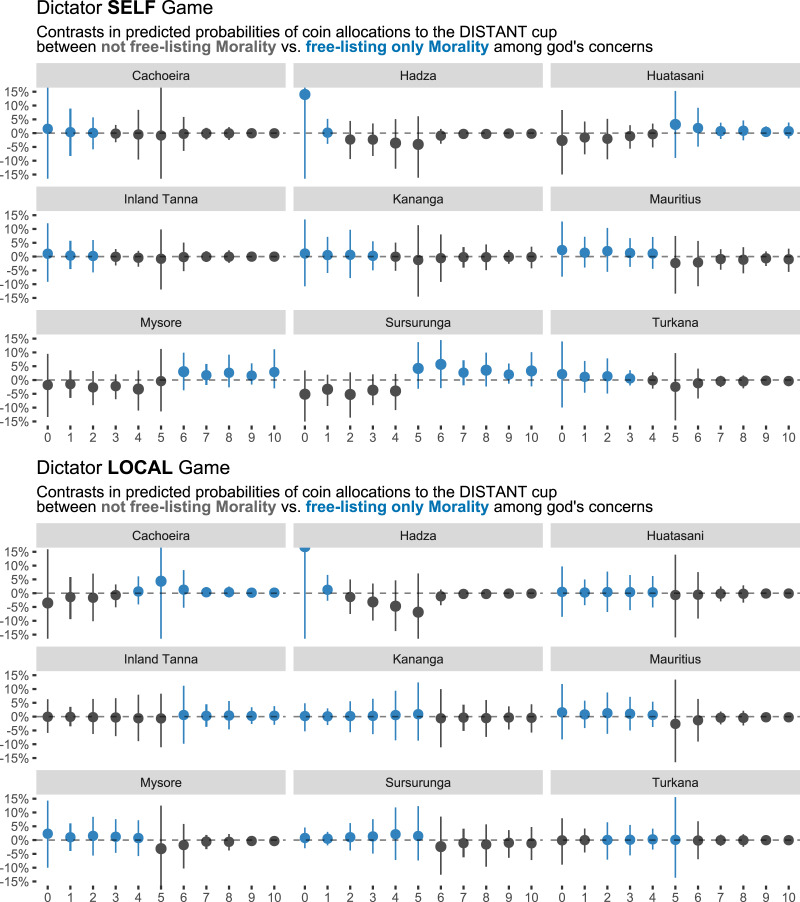
Dictator SELF (top) and LOCAL (bottom) Games. Marginal contrasts in posterior predicted probabilities of allocating 0–10 coins (*x*-axis) to the DISTANT cup (in percentage points). For a given number of coins, if the posterior mean contrast is positive (blue), there's an on-average higher probability of allocating that number of coins to the DISTANT cup, when free-listing only moral content (*M* = 1) among the focal deity's concerns. If the posterior mean contrast is negative (grey), there's an on-average higher probability of allocating that number of coins to the DISTANT cup, when not free-listing moral content (*M* = 0) among the focal deity's concerns. 0% (dashed line) means no difference. Points are posterior means and lines are 95% HPDIs.

Results are similar for the DGs. Figure 4 plots for the DGs (upper panel, SELF game; lower panel, LOCAL game) and for each site the contrast in posterior predicted probabilities of allocating 0–10 coins (*x*-axis) to the DISTANT cup when perceiving the focal deity as maximally moralistic (i.e. free-listing only moral responses, *M* = 1) compared with not moralistic at all (i.e. not free-listing any moral responses, *M* = 0). Blue points and lines imply, for any given number of coins, a positive posterior mean contrast such that participants were on average more likely to allocate this particular number of coins if they free-listed the focal deity as maximally moralistic.

If participants, who perceive the focal deity as moralistic, were more likely to be cooperative towards the DISTANT co-player, we would expect, then, a positive contrast (i.e. blue points and lines) for the higher coin allocations. While we do find this predicted relationship at a few sites (in the SELF game, Huatasani, Mysore, Sursurunga; and in the LOCAL game, Cachoeira, Coastal Tanna), at no sites are the results unequivocal: the posterior means are small in almost all cases. Hadza is the most striking exception; there, participants are predicted to be more likely to allocate no coins to the DISTANT cup on average if they free-list their focal deity as maximally moralistic – although note the wide and uncertain HPDIs.

As for the tight intervals around the higher coin allocations at several sites, these result from the cumulative nature of the models combined with a lower number of participants putting many coins in the DISTANT cup. That is, on the basis of these data, the models are quite confident that the differences in probability between listing only vs. not listing supernatural moral concerns are generally small for the higher coin allocations, because relatively few participants allocated many coins to the DISTANT cup (see also Figure 1).

To recap, then, even if some sites exhibit trends in the predicted direction, we fail to find reliable evidence overall of a clear positive relationship between perceiving a focal deity as moralistic and increased cooperation toward an anonymous, distant co-religionist. This inference did not change across various model specifications, as discussed in Section 4 (see also online supplementary materials, Section S5).

### 3.2. Interaction effect between gods’ punishment, knowledge and moral concerns

For H_2_, we assessed the regression coefficients of the interaction terms, their implied predictions on the probability scale as well as their model comparison metrics (see Section S2). While the main effects of punitiveness and, in particular, knowledge breadth were generally positive across model specifications (in line with Lang et al., 2019; Purzycki et al., 2016b), in no case did we find robust evidence for any interaction effect on any of the model comparison metrics. This is possibly owing in part to lack of sufficient statistical power (as also noted in the pre-registration), and in part to small, heterogeneous or non-existent effects. We nonetheless report from the interaction models here, since they are theoretically more informed. In any case, the interaction and additive models support qualitatively similar inferences, apart from slightly higher precision in estimates in the additive model.

## 4. **General** discussion

In this study, we assessed with individual-level data – across 15 diverse field sites and two permutations of each of two behavioural economic games – the importance of moral content in moralistic supernatural punishment beliefs and its role in motivating cooperative behaviour.

Specifically, we assessed (H_1_) whether free-listing a locally relevant moralistic deity as angered by breaches of morality predicts increased cooperation towards an anonymous, distant co-religionist. While some field sites exhibited trends in the predicted direction, overall we failed to find reliable evidence in favour of H_1_ across games and sites. This finding is consistent with previous studies (Lang et al., 2019; Purzycki et al., 2016b), which found that rating a relevant god as concerned with punishing theft, lying and murder does not reliably predict cooperation. We also tested (H_2_) for the presence of interaction effects with the focal deity's perceived degree of punitiveness and knowledge breadth; that is, whether the effect on economic game play of ascribing moral content was modified by these more general supernatural traits (reported in the online supplementary materials, see Section S5). While the main effects of punitiveness and, in particular, knowledge breadth were generally positive across model specifications (in line with Lang et al., 2019; Purzycki et al., 2016b), our study was inconclusive regarding their interactions, presumably owing to both insufficient statistical power and small, variable or non-existent effects.

In three sets of supplementary analyses, we explored a few additional questions (reported in the online supplementary materials, see Section S5). First, we assessed whether our main conclusions would change if instead of free-listed moral concern we used an index of moral concern measured with a set of scale items on particular moral offenses from Lang et al. (2019) and Purzycki et al. (2016b) (see Section S3.5). While neither Lang et al. (2019) nor Purzycki et al. (2016b) found a robust relationship between this ‘moral interest scale’ and the behavioural economic games, our statistical approach differed from theirs sufficiently to warrant a pre-registered re-analysis. Second, since the general free-list code ‘Morality’ captures responses that might not directly translate to the economic game contexts (e.g. ‘murder’, ‘violence’, etc.), we ran another set of analyses where we used a more narrow set of free-list responses as the main predictor. This set of free-list responses was preregistered to specifically pertain to resource and social exchange and therefore arguably have higher face validity in the context of the present study (see Section S3.4). Thus, the rationale for this analysis was to explore whether perceptions of supernatural punishment that are more relevant to the game contexts, compared with morality in general, more clearly predict cooperation in the games (see further discussion below). Third, we expanded the predictor of the main analysis such that it also included instances of the general free-list coding category ‘Virtue’, which overlaps conceptually with the ‘Morality’ coding category; a free-list response qualified as ‘Virtue’ if it satisfied the following: *individual qualities that may or may not have social ramifications* (e.g. hard-working, kind, bad conscience, etc.; see Bendixen et al., 2023a). While this latter analysis was not pre-registered, there is precedence in the published literature for lumping these two coding categories (e.g. Bendixen et al., 2023a; Purzycki and McNamara, 2016; Purzycki et al., 2016b). These supplementary analyses did not yield qualitatively different results than reported here.

What do we make of these results in the context of the family of theories on moralistic supernatural punishment beliefs reviewed at the outset? We first discuss a few methodological considerations before offering some more general reflections on this research program.

Note that in the SELF RAG (Figure 3, top), the more prominent positive effects appear when the probability of allocating coins to the DISTANT cup is generally lower (posterior means *<* 40% when *M* = 0; Hadza, Huatasani, Yasawa). With the RAG being a measure of fairness and impartial rule-following, it may be that supernatural moralistic beliefs are mostly effective at nudging people closer to playing fairly (e.g. getting closer to ~50% in the RAG) rather than making people overly cooperative (e.g. getting above 50% in the RAG). As for the LOCAL RAG (Figure 3, bottom), we see more diffuse effects. We speculate whether this could be due to a view that the morally ‘right’ thing to do is sometimes favouring a local co-religionist over a distant co-religionist – or at least that some participants and sites were guided by such intuition. These considerations emphasise a more general take-home point, namely that these kinds of data often call for site-specific contextualisation (see references to site-specific reports above). This inference is in line with the DG results, where we failed to capture a clear omnibus pattern across sites and game type (Figure 4). It's possible that unmodelled site-specific variation is more important when there are little-to-no game constraints, as is the case in the DG compared with the RAG.

Another possibility is that the artificial game set-ups do not map onto salient mental models of the deities in their relevant cultural contexts and that the games therefore fail to reflect relevant behavioural cues (cf. Cronk, 2007; Lightner et al., 2017). Recent work (Bendixen et al., 2023a; McNamara and Purzycki, 2020; Purzycki et al., 2022b) has emphasised that a fit between perceived supernatural concerns and particular socioecological dilemmas might be required for religious appeals and beliefs to have corresponding behavioural consequences. On that perspective, general moral infringements, as captured by our ‘Morality’ free-list code and by the ‘moral interest scale’ from Lang et al. (2019) and Purzycki et al. (2016b), might not be directly relevant to the experimental settings of the RAGs and DGs. To study religious systems in their culturally relevant context, rather than relying exclusively on standardised economic games, future empirical studies could evaluate this socioecological view by catering data collection to relevant local settings (Bendixen and Purzycki, 2023c), for instance through natural experiments (e.g. Atran et al., 2002), ethnographically salient games (e.g. Townsend et al., 2020) and vignette studies (e.g. Purzycki and Arakchaa, 2013).

To the extent that our methods do capture relevant mental and behavioural processes of moralistic supernatural punishment beliefs, our study – in line with and building on Lang et al. (2019) and Purzycki et al. (2016b) – suggests that supernatural moral concerns do not play a direct, cross-culturally reliable role in motivating cooperative actions, *above and beyond a deity's perceived capabilities for punishment and monitoring more generally*. Rather than driving behaviour directly, postulated supernatural concerns may instead make salient certain classes of collective action problems that religious systems adapt to – and perhaps address – through more indirect pathways (Bendixen and Purzycki, 2023a; Bendixen et al., 2023a; Purzycki et al., 2022b).

Indeed, many lines of evidence suggest that appeals to, beliefs about, and rituals directed towards the supernatural support social and ecological life-ways in diverse human societies (e.g. Bulbulia and Sosis, 2011; Leeson and Suarez, 2015; Rossano, 2007). However, above and beyond a few particularly focused studies (e.g. Ge et al., 2019; Lang et al., 2019; Power, 2017; Purzycki et al., 2016b; Townsend et al., 2020; Xygalatas et al., 2013), many ethnographically informed, individual-level findings remain primarily circumstantial (e.g. Atran et al., 2002; Bendixen et al., 2023a; Purzycki and Arakchaa, 2013; Singh et al., 2021). Future studies seeking to quantify the effect of supernatural punishment beliefs could build on these efforts by ensuring that the mental models and implied behavioural patterns under investigation map onto ethnographically salient and ecologically valid contexts (Bendixen and Purzycki, 2023c). This requires acute ethnographic attention to the constraints and affordances of the local socioecology as well as, through careful study design and statistical analysis, adhering to rigorous principles of contemporary causal inference.

## Research transparency and reproducibility

Pre-registration materials, supplementary analyses and plots, as well as data and analysis scripts for this study are openly available at: https://github.com/tbendixen/moral-freelist-econ.

## Supplementary material

For supplementary material accompanying this paper visit https://doi.org/10.1017/ehs.2023.15. click here to view supplementary material
