# Supplementary material for: Gods are watching and so what? Moralistic supernatural punishment across 15 cultures
Source: Evol Hum Sci. 2023 May 12;5:e18. doi: 10.1017/ehs.2023.15 (PMC10426076; doi:10.1017/ehs.2023.15)
Supplement: Supplementary file 1 [file S2513843X23000154sup001.pdf]

Supplementary Materials: Gods are watching and so what? Moralistic supernatural punishment across 15 cultures

Theiss Bendixen[a,*], Aaron D. Lightner[a], Coren Apicella[b], Quentin Atkinson[c,d], Alexander Bolyanatz[e], Emma Cohen[f], Carla Handley[g], Joseph Henrich[h], Eva Kundtová Klocová[i], Carolyn Lesorogol[j], Sarah Mathew[g], Rita A. McNamara[k], Cristina Moya[l], Ara Norenzayan[m], Caitlyn Placek[n], Montserrat Soler[o], Tom Vardy[p], Jonathan Weigel[q], Aiyana K. Willard[r], Dimitris Xygalatas[s,t], Martin Lang[i], Benjamin Grant Purzycki[a,*]

[a]*Aarhus University, DK*
[b]*Department of Psychology, University of Pennsylvania, USA*
[c]*Department of Psychology, University of Auckland, NZ*
[d]*Max Planck Institute for the Science of Human History, DE*
[e]*College of DuPage, USA*
[f]*Wadham College, University of Oxford, UK*
[g]*Arizona State University, USA*
[h]*Harvard University, USA*
[i]*LEVYNA, Masaryk University, CZ*
[j]*Washington University in St. Louis, USA*
[k]*School of Psychology, Victoria University of Wellington, NZ*
[l]*Department of Anthropology, University of California Davis, USA*
[m]*University of British Columbia, CA*
[n]*Department of Anthropology, Ball State University, USA*
[o]*Ob/Gyn and Women's Health Institute Cleveland Clinic, USA*
[p]*Department of International Development, London School of Economics, UK*
[q]*Haas School of Business, University of California Berkeley, USA*
[r]*Brunel University, UK*
[s]*Department of Anthropology, University of Connecticut, USA*
[t]*Department of Psychological Sciences, University of Connecticut, USA*

[*]Corresponding authors
Email addresses: tb@cas.au.dk (Theiss Bendixen), bgpurzycki@cas.au.dk (Benjamin Grant Purzycki)

Contents

S1 Causal Model **3**

S1.1 Causal identification assumptions 4

S2 Statistical models **5**

S2.1 RAG model 7

S2.1.1 Imputation models 9

S2.2 DG model 11

S2.3 *G*-computation 11

S3 Data **12**

S3.1 Covariates 12

S3.2 Free-list data cleaning 13

S3.3 Proportion vs. salience of moral free-list responses 13

S3.4 Game-relevant specific codes 14

S3.5 Moral Interest Scale 15

S4 Deviations from pre-registration protocol **15**

S5 Online supplementary plots and analyses **17**

S1. Causal Model

To guide model-building and inference and based on the above review of previous literature, Figure S1 illustrates our assumed causal structure of the data-generating process in the form of a *directed acyclic graph* (DAG; see Pearl et al., 2016).

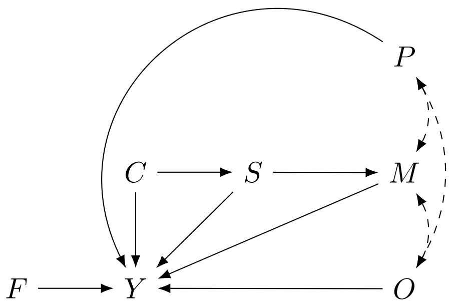

Figure S1: **Directed acyclic graph (DAG) of the assumed causal structure of the data-generating process**. C = number of children; S = food insecurity; P = punitive tendency of deity; O = knowledge breadth of deity; M = deity's degree of moral concern; Y = cooperation; F = Structural features of the game set-up. Dashed double-headed arrows refer to bidirectional causal relationships.

Here, Y denotes cooperation, M, P and O denote respectively the moral concern, punitive tendency, and knowledge breadth of a deity (between which we assume bidirectional causal relationships represented by dashed double-headed arrows), S denotes food (in)security, C denotes number of children, and F denotes a set of structural features of the game set-up (game order, game check, and game type; see Section S3.1). These assumed relationships derive from previous empirical studies and evidence syntheses[1] (Lang et al., 2019; Purzycki et al., 2016b, 2018a,b,c, 2022). Our target relationship, or *estimand*, then, is the direct effect $M \rightarrow Y$, where M is measured using free-lists and Y is operationalized as

[1]Note that our adjustment set is rather minimal. We justify this on the grounds that previous analyses (in particular, see Lang et al., 2019) did not find consistent relationships between common control variables (sex, education, etc.) and coin allocation to the DISTANT cup across a wide range of model specifications.

economic game play.

According to this causal model and assuming no relevant unobserved confounding, to block all back-door paths from M to Y, we need only adjust for P, O, and S. The path from C to M through S is blocked when we condition on S; conditioning on C is therefore not strictly necessary. However, including C can reduce variation in Y, thereby increasing the precision of the target relationship (see e.g., Cinelli et al., 2020, and references therein), and we therefore include both C and S in our conditioning set[2]. For the same reason, we include the structural features of the game set-up, F. The only colliders in this graph are the main predictor variables M, P and O, and the outcome variable, Y, and so, under this model, conditioning on covariates does not induce spurious associations in our estimand. Moreover, while we assume no systematic missingness pattern conditional on our covariates, our statistical models employ full Bayesian imputation of missing covariates (McElreath, 2020) in order not to discard data unnecessarily.

Next, we discuss the assumptions required for causal interpretation of our analysis.

S1.1. Causal identification assumptions

Our g-computation approach to estimation (Section S2.3) in an observational setting generally requires five key conditions for a causal interpretation of the main exposure (e.g., Hernan and Robins, 2020, ch. 13; Naimi et al., 2017).

We assume that the potential outcomes (coin allocations in the behavioral games) under varying levels of exposure (free-listed Morality) are independent from the observed outcomes (*conditional exchangeability*). In a perfectly randomized trial, this is the case since randomization ensures that the probability of treatment is independent of the outcome – we say that the treatment and control groups are "exchangeable". However, in an observational

[2]Two additional variables could be included on these grounds, namely measures of emotional closeness to the LOCAL/DISTANT players using pictorial "fusion" scales. While previous research found small effects of these variables on impartial coin allocations, a concern was raised that rather than being measures of "fusion" *per se*, these instruments might measure prosocial tendencies in general (cf., Purzycki and Lang, 2019). Given ambiguities around validity, we refrain from including these variables here.

setting, there can be countless factors that both influence exposure levels and the outcome. It's a main goal of our statistical model to adjust for these confounding factors, in order to obtain *conditional* exchangeability. We used a DAG (Figure S1) to guide our statistical adjustment. In essence, we sought to statistically adjust for all causal paths that both influence our exposure M and outcome Y of interest.

Relatedly, we assume *no model misspecification*, which entails that our model is specified correctly (e.g., in terms of functional relationships, no omitted confounding variables, etc.). However, whether any given statistical model and adjustment sets are sufficient to ensure these conditions hold is generally not empirically testable. Similarly, we assume that our variables are *measured without error*, another difficult-to-verify assumption.

We further assume that an individual's observed outcome under a given exposure is equivalent to the potential outcome that would've been observed under that exposure (*counterfactual consistency*). In other words, in the context of our g-computation procedure, when we obtain expected values for participants setting $M = m$, we assume that we obtain the values that we in fact would've observed, if those participants had been observed under $M = m$.

Finally, we assume *positivity*, which implies that individuals have similar exposure levels within all confounder levels. While this is empirically unlikely to hold in our case (in that we have several covariates, including a continuous on (i.e., number of children) as well as several groups), lack of positivity can be ignored to the extent that we're willing to assume that estimates for the strata with zero observations can be extrapolated from the model fitted on the observed strata (Hernan and Robins, 2020, p. 162). To that effect, our Bayesian multilevel models borrow information across clusters to inform, impute, and adaptively regularize estimates of all other clusters.

S2. Statistical models

In this section, we lay out our statistical approach and detail the main models in formal notation. The models are extensions of the main model from Purzycki et al. (2018b). We

analyzed the RAG with a binomial model (Section S2.1) and the DG with an ordered categorical model (Section S2.2) and model the SELF and LOCAL games in separate models. Both sets of models include varying effects to manage repeated observations on groups. We ran the models on simplified simulated data to ensure that the models in fact recover key parameters in an ideal scenario under the assumed data-generating process. Key model diagnostics and posterior predictive checks were generally acceptable and are reported in the online supplementary materials (Section S5).

For H_1 the key parameter is that of free-listed morality β^{M} on coin allocation y. To assess H_2 for each of the two outcomes we fit two different models: (1) The theoretically informed "interaction model" including three two-way interaction terms, one for punitiveness and free-listed morality β^{MP}, another for knowledge breadth and free-listed morality β^{MO}, a third for punitiveness and knowledge breadth β^{PO} (not directly relevant to H_2 but a sub-component of the three-way interaction), and one three-way interaction term β^{MPO}, as well as main effects of morality β^{M}, punitiveness β^{P} and knowledge breadth β^{O}; and (2) an "additive model", which excludes all interaction terms but retains the main effects, and serves then as a "null model" in contrast to the theoretically informed interaction model.

We then 1) inspected the interaction terms to evaluate whether their coefficients have the bulk of their mass above a log odds of 0 (i.e., "no effect"), 2) assessed the posterior predictions of the interaction terms on their natural scales, and 3) compared the two model pairs with approximate leave-one-out cross-validation (LOO-CV; Vehtari et al., 2017), a convenient model comparison tool that computes metrics of out-of-sample predictive accuracy while penalizing model complexity[3]. Model code and data were prepared with the `rethinking` package (McElreath, 2020) for `R` and fit with `Stan` via `cmdstanr` (Carpenter et al., 2017;

[3]More specifically, LOO-CV approximates the focal models' relative accuracy in predicting a new observation in one of the observed groups. An alternative approach is leave-one-group out cross-validation (LOGO-CV) which assesses models' relative accuracy in predicting a new observation in a new (unobserved) group. This procedure is much more computationally intensive, because it involves refitting the model k times, where k equals the number of observed groups. For this reason, and because we're here mostly interested in global, cross-cultural – in contrast to site-specific – inferences, we refrain from pursuing LOGO-CV in the present analysis.

Gabry et al., 2022).

Below, we explain the full interaction models for each game in pieces.

S2.1. RAG model

To model the coin allocations to the distant cup y out of a total of 30 coins in the RAG, we use Bayesian multilevel binomial regression:

$$
\begin{aligned}
y_i &\sim \text{Binomial}(30, p_i) && (1)\\
\text{logit}(p_i) &= \alpha + a_{\text{group}[i]} && (2)\\
&+ \beta^{\text{M}}_{\text{group}[i]} M_i + \beta^{\text{P}}_{\text{group}[i]} P_i + \beta^{\text{O}}_{\text{group}[i]} O_i && (3)\\
&+ \beta^{\text{MP}}_{\text{group}[i]} M_i P_i + \beta^{\text{MO}}_{\text{group}[i]} M_i O_i + \beta^{\text{PO}}_{\text{group}[i]} P_i O_i && (4)\\
&+ \beta^{\text{MPO}}_{\text{group}[i]} M_i P_i O_i && (5)\\
&+ \beta^{\text{children}} c_i + \beta^{\text{food}} s_i && (6)\\
&+ \beta^{\text{order}} r_i + \beta^{\text{check}} \chi_i && (7)
\end{aligned}
$$

The linear model $\text{logit}(p_i)$ (line 2) includes an average intercept α and varying intercepts group (i.e., field site). The next lines (3-5) captures the three cultural variables of interest: free-listed moral code M, gods' punishment P, and knowledge breadth (i.e., omniscience) O and their respective interactions. For these parameters, each group has its own varying slope. The last two lines contain simple (i.e., fixed) individual-level effects for the remaining conditioning set: number of children, food insecurity, game order and game check, respectively.

All simple effects above are assigned weakly-regularizing priors, $\text{Normal}(0, 1)$. These guard against finding strong effects in small samples or those that vary considerably in responses, but are easily overwhelmed in large or consistent samples. The varying effects for group are bound together in a common variance-covariance matrix:

$$\begin{bmatrix} a_{\text{group}} \\ \beta^{\text{M}}_{\text{group}} \\ \beta^{\text{P}}_{\text{group}} \\ \beta^{\text{O}}_{\text{group}} \\ \beta^{\text{MP}}_{\text{group}} \\ \beta^{\text{MO}}_{\text{group}} \\ \beta^{\text{PO}}_{\text{group}} \\ \beta^{\text{MPO}}_{\text{group}} \end{bmatrix} \sim \text{MVNormal}\left(\begin{bmatrix} 0 \\ \beta^{\text{M}} \\ \beta^{\text{P}} \\ \beta^{\text{O}} \\ \beta^{\text{MP}} \\ \beta^{\text{MO}} \\ \beta^{\text{PO}} \\ \beta^{\text{MPO}} \end{bmatrix}, \mathbf{SRS} \right)$$

where $\mathbf{S}$ is a diagonal matrix of standard deviations of the intercept and the main and interaction terms for the three cultural variables of interest:

$$\mathbf{S} = \begin{bmatrix} \sigma_a & 0 & 0 & 0 & 0 & 0 & 0 & 0 \\ 0 & \sigma_{\beta^{\text{M}}} & 0 & 0 & 0 & 0 & 0 & 0 \\ 0 & 0 & \sigma_{\beta^{\text{P}}} & 0 & 0 & 0 & 0 & 0 \\ 0 & 0 & 0 & \sigma_{\beta^{\text{O}}} & 0 & 0 & 0 & 0 \\ 0 & 0 & 0 & 0 & \sigma_{\beta^{\text{MP}}} & 0 & 0 & 0 \\ 0 & 0 & 0 & 0 & 0 & \sigma_{\beta^{\text{MO}}} & 0 & 0 \\ 0 & 0 & 0 & 0 & 0 & 0 & \sigma_{\beta^{\text{PO}}} & 0 \\ 0 & 0 & 0 & 0 & 0 & 0 & 0 & \sigma_{\beta^{\text{MPO}}} \end{bmatrix}$$

and $\mathbf{R}$ is a full rank correlation matrix of the same variables. Each standard deviation is assigned an independent Exponential(1) prior as before, and $\mathbf{R}$ is given a weakly regularizing prior from the LKJ family (Lewandowski et al., 2009), a common choice of prior distribution for covariance matrices:

$$\mathbf{R} \sim \text{LKJCorr}(4)$$

S2.1.1. Imputation models

Our three key predictor variables above have missing values: free-listed morality, punishment, and knowledge breadth. We build an imputation model for each of these variables so that the imputed values are informed by their field site. For example, the morality free-list imputation model looks as follows:

$$\begin{aligned}
M_i &\sim \text{Normal}(\mu_i^{\text{m}}, \sigma^{\text{m}}) \\
\mu_i^{\text{m}} &= M_{\text{group}[i]} \\
\sigma^{\text{m}} &\sim \text{Exp}(1) \\
M_{\text{group}} &\sim \text{Normal}(\mu^{\text{M}}, \sigma^{\text{M}}) \\
\mu^{\text{M}} &\sim \text{Normal}(0.5, 0.5) \\
\sigma^{\text{M}} &\sim \text{Exp}(1)
\end{aligned}$$

where the imputed values M range between 0 and 1 and are drawn from a normal distribution with mean μ^{m} and standard deviation σ^{m} that are informed by the individual's field site's estimated mean proportion of free-listed moral items M_{group}. This value is in turn drawn from a normal distribution with a mean and standard deviation that are assigned weakly regularizing priors themselves. The prior for μ^{M}, the mean proportion of free-listed moral items for a given group, is centered on 0.5 as it is the mid-point of the variable, which ranges between 0 and 1; however, with a standard deviation of 2 this estimate is allowed to take on a wide range of values, although we constrain it via `Stan` at 0 and 1. The imputation models for punishment and knowledge breadth are similar, although those two variables are each means of two binary items and can therefore take on values 0, 1, and 2[4].

Similarly, following the same structure, we impute missing values in number of children so that the imputations are informed by the respective site-specific mean number of children

[4] In the accompanying `R` scripts, we show how to plot the distribution of imputed values against the observed data.

(truncated at zero).

$$\begin{aligned}
\text{children}_i &\sim \text{Normal}(\text{children}_i^{\mu}, \text{children}_{\sigma}) \\
\text{children}_i^{\mu} &= \text{Children}_{\text{group}[i]} \\
\text{children}_{\sigma} &\sim \text{Exp}(10) \\
\text{Children}_{\text{group}} &\sim \text{Normal}(\mu^{\text{Children}}, \sigma^{\text{Children}}) \\
\mu^{\text{Children}} &\sim \text{Normal}(1, 2) \\
\sigma^{\text{Children}} &\sim \text{Exp}(1)
\end{aligned}$$

We relied on the following prior distributions for the remaining parameters:

$$\begin{aligned}
\text{food} &\sim \text{Bernoulli}(\phi_{\text{food}}) \\
\phi_{\text{food}} &\sim \text{Beta}(1, 1) \\
\text{order} &\sim \text{Bernoulli}(0.5) \\
\text{check} &\sim \text{Bernoulli}(\phi_{\text{check}}) \\
\phi_{\text{check}} &\sim \text{Beta}(1, 1)
\end{aligned}$$

The sub-models for the food and check variables impute missing values (there are no missing values in the order variable) using Bayesian inference. Since these two variables are binary (0/1), we say that they are drawn from a Bernoulli distribution with probability ϕ, which is estimated from the sample and given a weakly regularizing beta prior. When we plot the imputations of the ϕ_{food} and ϕ_{check} parameters (see Section S5), note that these pertain to the posterior probabilities of a 1 and not the actual, imputed binary values.

S2.2. DG model

To model coin allocations to the distant cup y out of a total of 10 coins in the DG, we use an ordered categorical likelihood model, where we regard each coin as a discrete ordered response

$$y_i \sim \text{Ordered Categorical}(\eta_i, K)$$

The cumulative property of the ordered categorical neatly captures the cumulative aspect of coin allocations in the DG game. However, since zero is not a valid value for the ordered categorical model but was an option in the game (i.e., no coins allocated to the distant cup) and falls naturally on the ordering of the response (i.e., zero coins is less than one, less than two, etc.), we add a "dummy coin" to the response variable, such that the response value ranks from one to 11, where one means zero coins, two means one coin, etc. This yields a vector of $11 - 1 = 10$ random cut-points K, on which we put a prior of $\text{Normal}(0, 2)$ to be estimated along with a linear model η_i that is otherwise similar to the RAG model.

S2.3. G-computation

Once the models are fitted, the marginal contrasts are obtained as follows[5]:

1. Duplicate original dataset and set $M = 0$ (i.e., no moral items in an individual's free-list) for all individuals, retaining covariates as observed.
2. Duplicate original dataset and set $M = 1$ (i.e., only moral items in an individual's free-list) for all individuals, retaining covariates as observed.
3. For each draw of the posterior distribution, use the fitted model to get expected values for each individual in each of these counter-factual datasets.

[5]This procedure is commonly referred to as *g-computation* or *standardisation* (see e.g., Hernan and Robins, 2020, ch. 13). With it, we aim for a target quantity akin to a marginal "average treatment effect" using an "observed-values" approach (e.g., Hanmer and Ozan Kalkan, 2013). For individuals with missing covariates, we get predictions at the posterior mean of the imputed values; see Morris et al. (2022) for some discussion (although in a clinical trial context) on using mean imputation for missing covariate data when applying standardisation.

4. Within each draw of the posterior distribution, compute the contrast in predictions between the two focal conditions, $M = 1$ and $M = 0$. That is, the contrast of interest is given by $\mathrm{E}[Y^{M=1,Z=z}] - \mathrm{E}[Y^{M=0,Z=z}]$, where Y is the predicted outcome, and the superscripts $Z = z$ denote that covariates Z are averaged over at their observed values z using the expectation operator $\mathrm{E}[.]$.
5. Finally, for each site, we summarize the contrasts across all posterior draws by their posterior mean and 95% highest posterior density interval (HPDI), the narrowest region of the posterior distribution containing 95% of the parameter estimates[6].

S3. Data

S3.1. Covariates

A comprehensive overview of variables, sampling procedures, and field sites characteristics employed in the Evolution of Religion and Morality Project can be found in Purzycki et al. (2016a) and Lang et al. (2019). Here, we describe the operationalization of covariates relevant for the present study. In addition to the conditioning set identified in Section S1, we include two variables (game order and game check; see explication below) related to the structural features of the game set-up (see Purzycki et al., 2018b).

Children: the self-reported number of children that a participant has fathered or given birth to.

Food insecurity: participant's self-reported food or material (in)security measured with a dichotomous (yes/no) item: "Do you worry that in the next month your household will have a time when it is not able to buy or produce enough food to eat?".

Punitiveness: The mean of two dichotomous (yes/no) items: "Does [moralistic deity] ever punish people for their behavior?" and "Can [moralistic deity] influence what happens to people after they die?".

[6] Another way to think of an HPDI is that all estimates within the HPDI have higher density than any other estimate outside of it.

Knowledge breadth: The mean of two dichotomous (yes/no) items: "Can [moralistic deity] see into people's hearts or know their thoughts and feelings?" and "Can [moralistic deity] see what people are doing if they are far away in [a distant town or city familiar to locals]?"

Game order: An indicator denoting which game participants played first. 0 = SELF Game first, 1 = LOCAL Game first.

Game check: An indicator denoting whether, when asked what they thought the games were about during debriefing, a participant's response included (= 1) "honesty," "fairness," and/or "cheating".

S3.2. Free-list data cleaning

Following the workflow of Bendixen et al. (2023), the data entries for the general codes were cleaned and systematized (e.g., in terms of spelling, typos, and blank spaces) to avoid that the same codes are treated as separate by the parsing in `R` and `AnthroTools`. Then, in cases of disagreement between coders, we selected for final analysis the best fitting of the two codes according to the general coding scheme (see Purzycki and McNamara, 2016). A column designating these (dis)agreements was added to the spreadsheet, and the selected code was stored in a separate column for full transparency.

S3.3. Proportion vs. salience of moral free-list responses

As we are interested in measuring the extent to which individuals ascribe moral concern to their deities, we use the *proportion of moral items* in each participant's lists as our focal predictor for all main models. We take the proportion of moral items to deal with multiple listings of moral responses.

A popular alternative is a *salience score*. A salience score is computed by subtracting an item's order number, k, from 1 plus the total number of items a participant listed. This number is then divided by the total number of items listed, $\frac{n+1-k}{n}$. All items listed first thus get an item salience of 1. Items listed earlier are typically easier to access or recall, and thus constitute a form of *cognitive* salience.

For the current study, we use proportion rather than salience for two main reasons. First, based on previous analyses (Bendixen and Purzycki, 2023; Bendixen et al., 2023), we expected that salience scores would produce ceiling effects, such that many participants list moral items as the first response, in turn yielding little variation in the variable. Second, whereas salience scores are generally considered a measure of recall (e.g., the ease with which an association comes to mind), we consider proportion to be a more appropriate measure of the *degree* to which participants perceive their deity as moralistic, a more apt measure for present purposes.

S3.4. Game-relevant specific codes

In the pre-registration document (p. 25-26), we wrote: "Since the general free-list code "Morality" captures responses that might not directly translate to the economic game contexts (e.g., "murder", "violence", etc.), we're also going to code for a more narrow set of free-list responses with a higher face validity for the present study having to do specifically with resource and social exchange (e.g., dishonesty, insincerity, not trustworthy, unfairness, lies, stealing, deception, greed, no sharing, selfishness; along with their synonyms and derivations)."

Based on the available codes and responses, we ended up coding for the presence of the following responses: "Dishonesty", "Dishonest", "Deception", "Truthfulness.n", "Not Truthful", "Helpful.n", "Lies", "Stealing", "Theft", "Greed", "Selfish", "Not Sharing", "Egoism", "Cooperation.n", "Kindness.n", "Justice.n", "Unjustice", "Exploiting Others", "Compassion.n", "Betray", "Betrayal", "Loyalty.n", "Loyal.n", "Disloyal", "Disloyalty", where the ".n" suffix was one coder's idiosyncratic way of indicating a negative (e.g., "Truthfulness.n" indicates that the moralistic god is angered by untruthfulness).

We recorded a "1" if any of these codes figured in either of the two coders' columns and then, for each participant, took the proportion of these responses to the total number of responses listed, similar to the main analysis.

S3.5. Moral Interest Scale

The moral interest scale took the form: *How important is it for* [moralistic deity] *to punish*: [theft/lying/murder]? Responses were on scales of 0 to 4: (0) Not important at all; (1) A little important; (2) Important; (3) Very important; and (4) The most important. Responses are then averaged with missing values dropped.

One issue with this approach is that it assumes that, in cases of missing responses, participants are consistent across the three items—e.g., a participant can get a maximum score on the index either by selecting (4) on all three items or on just one or two of the items, if failing to respond to the remaining item(s). Another issue is the lack of uncertainty around the index score. We use the scale as described here, to be consistent with previous research (Lang et al., 2019; Purzycki et al., 2016b).

S4. Deviations from pre-registration protocol

Here, we document the deviations we took from the pre-registered statistical protocol. Deviations were minor in nature, and they were all due to a combination of oversight and unforeseen practical complications.

- In contrast to the pre-registration, we ended up modeling the SELF and LOCAL game outcomes in separate models. The reason for this was mainly computational. Modeling the SELF and LOCAL games in the same model required by-participant random intercepts to accommodate repeated observations on participants across game types. However, the by-participant random intercepts made our LOO model comparison unstable, due to some observations being too influential in the importance sampling procedure. We therefore dropped the by-participant random intercepts.
- We tightened the priors from $\text{Normal}(0.5, 2)$ to $\text{Normal}(0.5, 0.5)$ on μ^{M} and from $\text{Normal}(1, 2)$ to $\text{Normal}(0.5, 0.5)$ on μ^{P} and μ^{O}. These parameters constrain the group mean imputation of missing values in M, P and O, respectively. The pre-registered priors for μ^{P} and μ^{O} were constructed on the basis of these variables taking on values

0-2; however, they in fact range from 0-1. The relatively wide standard deviation of the prior for μ^{M} was likewise an oversight.

- Model parameters `bMavg`, `bPavg`, and `bOavg` in the pre-registered model code had priors but are redundant and are therefore deleted.
- We made a coding error in the imputation routine for number of children C. The pre-registered line `C_mu <- C[group]` was therefore changed to `C_mu <- Cavg[group]`.
- We deleted the imputation sub-models for the order and type variables, since there are no missing values in these variables in the dataset.
- We removed range constraints on `S_impute` and `check_impute`, since constraints are redundant (these parameters are already constrained by the beta distribution used for imputation).
- We pre-registered the supplementary analysis of the "moral interest" scale and specified that we'd transform the scale such that it ranged from 0-1. However, we retained it as used in Lang et al. (2019), such that it ranges from 0-4 and modified the prior used for imputation accordingly. Further, since Hadza participants answered these items on a binary scale, they are excluded from the analysis.
- In an exploratory (not pre-registered) supplementary analysis, we expanded the predictor of the main analysis such that it also included instances of the general free-list coding category "Virtue", which overlaps conceptually with the "Morality" coding category. A free-list response qualifies as "Virtue" if it satisfies the following: *individual qualities that may or may not have social ramifications* (e.g., hard-working, kind, bad conscience, etc.) (see Bendixen et al., 2023). Indeed, there's precedence in the published literature for lumping these two coding categories (e.g., Bendixen et al., 2023; Purzycki and McNamara, 2016; Purzycki et al., 2016b). However, it yielded similar results as reported in the main manuscript.
- We pre-registered a set of supplementary analysis whereby we'd fit an ordered beta model to the Dictator Game data. However, due to very reasonable posterior pre-

dictive fit of the ordinal model to the Dictator Game data, we refrained from fitting corresponding ordered beta models.

S5. Online supplementary plots and analyses

At this study's Git repo (`https://github.com/tbendixen/moral-freelist-econ`), we report supplementary plots and analyses in separate notebooks, for the sake of compact overview:

- `model_fits.pdf` reports coefficient plots, trace rank plots, and posterior predictive checks for all models as well as some simple diagnostics (i.e., number of divergent transitions) and model comparison metrics.
- `rag_results_plots.pdf` reports plots for all supplementary models of the Random Allocation Game.
- `dg_results_plots.pdf` reports plots for all supplementary models of the Dictator Game.
- `imp_plots.pdf` reports plots from missing data imputations of all variables and across all main models.

For reference, Table S1 provides an overview of all model specifications.

Table S1: **Overview of all model specifications**. Models above the dashed line are the "main models"; below are the "supplementary models". Game-specific sample sizes: RAG SELF $N = 1033$; RAG LOCAL $N = 1028$; DG SELF $N = 1077$; DG LOCAL $N = 1066$.

Model name	Game	Morality measure, M	SELF/LOCAL	Model type
m_rag_self_int	RAG	Free-listed Morality	SELF	Interaction
m_rag_local_int	RAG	Free-listed Morality	LOCAL	Interaction
m_rag_self_add	RAG	Free-listed Morality	SELF	Additive
m_rag_local_add	RAG	Free-listed Morality	LOCAL	Additive
m_dg_self_int	DG	Free-listed Morality	SELF	Interaction
m_dg_local_int	DG	Free-listed Morality	LOCAL	Interaction
m_dg_self_add	DG	Free-listed Morality	SELF	Additive
m_dg_local_add	DG	Free-listed Morality	LOCAL	Additive
m_rag_index_self_int	RAG	Moral Interest Scale	SELF	Interaction
m_rag_index_local_int	RAG	Moral Interest Scale	LOCAL	Interaction
m_rag_index_self_add	RAG	Moral Interest Scale	SELF	Additive
m_rag_index_local_add	RAG	Moral Interest Scale	LOCAL	Additive
m_dg_index_self_int	DG	Moral Interest Scale	SELF	Interaction
m_dg_index_local_int	DG	Moral Interest Scale	LOCAL	Interaction
m_dg_index_self_add	DG	Moral Interest Scale	SELF	Additive
m_dg_index_local_add	DG	Moral Interest Scale	LOCAL	Additive
m_rag_spec_self_int	RAG	Game-relevant code	SELF	Interaction
m_rag_spec_local_int	RAG	Game-relevant code	LOCAL	Interaction
m_rag_spec_self_add	RAG	Game-relevant code	SELF	Additive
m_rag_spec_local_add	RAG	Game-relevant code	LOCAL	Additive
m_dg_spec_self_int	DG	Game-relevant code	SELF	Interaction
m_dg_spec_local_int	DG	Game-relevant code	LOCAL	Interaction
m_dg_spec_self_add	DG	Game-relevant code	SELF	Additive
m_dg_spec_local_add	DG	Game-relevant code	LOCAL	Additive
m_rag_mv_self_int	RAG	Free-listed Morality + Virtue	SELF	Interaction
m_rag_mv_local_int	RAG	Free-listed Morality + Virtue	LOCAL	Interaction
m_rag_mv_self_add	RAG	Free-listed Morality + Virtue	SELF	Additive
m_rag_mv_local_add	RAG	Free-listed Morality + Virtue	LOCAL	Additive
m_dg_mv_self_int	DG	Free-listed Morality + Virtue	SELF	Interaction
m_dg_mv_local_int	DG	Free-listed Morality + Virtue	LOCAL	Interaction
m_dg_mv_self_add	DG	Free-listed Morality + Virtue	SELF	Additive
m_dg_mv_local_add	DG	Free-listed Morality + Virtue	LOCAL	Additive

References

Bendixen, T., Apicella, C., Atkinson, Q., Cohen, E., Henrich, J., McNamara, R. A., Norenzayan, A., Willard, A. K., Xygalatas, D., and Purzycki, B. G. (2023). Appealing to the minds of gods: Religious beliefs and appeals correspond to features of local social ecologies. *Religion, Brain & Behavior*.

Bendixen, T. and Purzycki, B. G. (2023). Cognitive and cultural models in psychological science: A tutorial on modeling free-list data as a dependent variable in Bayesian regression. *Psychological Methods*.

Carpenter, B., Gelman, A., Hoffman, M., Lee, D., Goodrich, B., Betancourt, M., Brubaker, M., Guo, J., Li, P., and Riddell, A. (2017). Stan: A probabilistic programming language. *Journal of Statistical Software*, 76(1).

Cinelli, C., Forney, A., and Pearl, J. (2020). A crash course in good and bad controls. *Sociological Methods & Research*, page 00491241221099552.

Gabry, J., Cešnovar, R., and Johnson, A. (2022). *cmdstanr: R Interface to 'CmdStan'*. https://mc-stan.org/cmdstanr/, https://discourse.mc-stan.org.

Hanmer, M. J. and Ozan Kalkan, K. (2013). Behind the curve: Clarifying the best approach to calculating predicted probabilities and marginal effects from limited dependent variable models. *American Journal of Political Science*, 57(1):263–277.

Hernan, M. and Robins, J. (2020). Causal inference: What if?

Lang, M., Purzycki, B. G., Apicella, C. L., Atkinson, Q. D., Bolyanatz, A., Cohen, E., Handley, C., Kundtová Klocová, E., Lesorogol, C., Mathew, S., et al. (2019). Moralizing gods, impartiality and religious parochialism across 15 societies. *Proceedings of the Royal Society B*, 286(1898):20190202.

Lewandowski, D., Kurowicka, D., and Joe, H. (2009). Generating random correlation matrices based on vines and extended onion method. *Journal of multivariate analysis*, 100(9):1989–2001.

McElreath, R. (2020). *Statistical Rethinking: A Bayesian course with examples in R and Stan*. CRC Press, second edition.

Morris, T. P., Walker, A. S., Williamson, E. J., and White, I. R. (2022). Planning a method for covariate adjustment in individually randomised trials: a practical guide. *Trials*, 23(1):1–17.

Naimi, A. I., Cole, S. R., and Kennedy, E. H. (2017). An introduction to g methods. *International journal of epidemiology*, 46(2):756–762.

Pearl, J., Glymour, M., and Jewell, N. P. (2016). *Causal inference in statistics: A primer*. John Wiley & Sons.

Purzycki, B. G., Apicella, C., Atkinson, Q. D., Cohen, E., McNamara, R. A., Willard, A. K., Xygalatas, D., Norenzayan, A., and Henrich, J. (2016a). Cross-cultural dataset for the evolution of religion and morality project. *Scientific Data*, 3:160099.

Purzycki, B. G., Apicella, C., Atkinson, Q. D., Cohen, E., McNamara, R. A., Willard, A. K., Xygalatas, D., Norenzayan, A., and Henrich, J. (2016b). Moralistic gods, supernatural punishment and the expansion of human sociality. *Nature*, 530(7590):327–330.

Purzycki, B. G., Henrich, J., Apicella, C., Atkinson, Q. D., Baimel, A., Cohen, E., McNamara, R. A., Willard, A. K., Xygalatas, D., and Norenzayan, A. (2018a). The evolution of religion and morality: a synthesis of ethnographic and experimental evidence from eight societies. *Religion, Brain & Behavior*, 8(2):101–132.

Purzycki, B. G. and Lang, M. (2019). Identity fusion, outgroup relations, and sacrifice: A cross-cultural test. *Cognition*, 186:1–6.

Purzycki, B. G., Lang, M., Henrich, J., and Norenzayan, A. (2022). The evolution of religion and morality: Reflections and looking ahead. *Religion, Brain & Behavior*, X.

Purzycki, B. G. and McNamara, R. A. (2016). An ecological theory of gods' minds. In De Cruz, H. and Nichols, R., editors, *Cognitive Science of Religion and Its Philosophical Implications*, pages 143–167. Continuum, New York.

Purzycki, B. G., Pisor, A., Apicella, C., Atkinson, Q. D., Cohen, E., Henrich, J., McElreath, R., McNamara, R. A., Norenzayan, A., Willard, A. K., and Xygalatas, D. (2018b). The cognitive and cultural foundations of moral behavior. *Evolution and Human Behavior*, 39(5):490–501.

Purzycki, B. G., Ross, C. T., Apicella, C., Atkinson, Q. D., Cohen, E., McNamara, R. A., Willard, A. K., Xygalatas, D., Norenzayan, A., and Henrich, J. (2018c). Material security, life history, and moralistic religions: A cross-cultural examination. *PloS one*, 13(3):e0193856.

Vehtari, A., Gelman, A., and Gabry, J. (2017). Practical Bayesian model evaluation using leave-one-out cross-validation and WAIC. *Statistics and Computing*, 27(5):1413–1432.
